# Lifetime Number of Mates Interacts with Female Age to Determine Reproductive Success in Female Guppies

**DOI:** 10.1371/journal.pone.0047507

**Published:** 2012-10-12

**Authors:** Jonathan P. Evans

**Affiliations:** Centre for Evolutionary Biology, School of Animal Biology, University of Western Australia, Nedlands, Western Australia, Australia; California State University Fullerton, United States of America

## Abstract

In many species, mating with multiple males confers benefits to females, but these benefits may be offset by the direct and indirect costs associated with elevated mating frequency. Although mating frequency (number of mating events) is often positively associated with the degree of multiple mating (actual number of males mated), most studies have experimentally separated these effects when exploring their implications for female fitness. In this paper I describe an alternative approach using the guppy *Poecilia reticulata*, a livebearing freshwater fish in which females benefit directly and indirectly from mating with multiple males via consensual matings but incur direct and indirect costs of mating as a consequence of male sexual harassment. In the present study, females were experimentally assigned different numbers of mates throughout their lives in order to explore how elevated mating frequency and multiple mating combine to influence lifetime reproductive success (LRS) and survival (i.e. direct components of female fitness). Under this mating design, survival and LRS were not significantly affected by mating treatment, but there was a significant interaction between brood size and reproductive cycle (a correlate of female age) because females assigned to the high mating treatment produced significantly fewer offspring later in life compared to their low-mating counterparts. This negative effect of mating treatment later in life may be important in these relatively long-lived fishes, and this effect may be further exacerbated by the known cross-generational fitness costs of sexual harassment in guppies.

## Introduction

Female multiple mating, where females mate with two or more males within a single reproductive episode, is taxonomically widespread among sexually reproducing animals [1]. Over the past few decades, a large body of theoretical and empirical research has focussed on the underlying evolutionary basis for female multiple mating, and in particular the potential benefits that females accrue from mating with more than one male during a single reproductive event [2]–[5]. While much of this work reveals that females can obtain either direct (e.g. fecundity-enhancing) or indirect (genetically-transmitted) benefits by mating with multiple males [6], [7], there is also accumulating evidence that conflicts of interest between the sexes over optimal mating rates can result in fitness costs, rather than benefits, for multiply-mated females [8], [9]. For example, experiments on insects have revealed that elevated mating frequency can significantly reduce a female's lifetime fecundity when the number of mates is held constant [10], while recent work on songbirds suggests that there may be indirect selection *against* female extra-pair reproduction [11].

Studies of female multiple mating that generate estimates of lifetime reproductive success (LRS) are useful because they also consider potential costs associated with elevated mating rates that may be specific to particular phases of a female's lifespan. For example, females with relatively high mating rates can suffer reductions in longevity [12] and earlier onset of reproductive senescence [13]. Such costs may be missed in cross-sectional studies that focus on components of female reproductive success at a specific point in time. Among studies that have generated estimates of LRS when assessing the benefits of female multiple mating, there is evidence that the direct benefits can be sufficient to outweigh any direct costs of mating [14]–[16]. Nevertheless, such studies are relatively scarce, and not all of the available evidence from LRS studies supports the hypothesis that multiple mating is selectively advantageous for females [17].

The guppy *Poecilia reticulata* is a sexually promiscuous livebearing fish that exhibits some of the highest recorded levels of multiple mating of any vertebrate [18]–[20]. Guppies are relatively long-lived, with females exhibiting pre- and post-reproductive lifespans that are comparable to those of birds and mammals [21]. During their brief sexually receptive phase each month (*ca*. 1–2 days after producing a brood), females solicit copulations from multiple males [22], which in turn confers several benefits, including the production of more and larger offspring exhibiting enhanced predator evasion capabilities [23], [24] and greater fecundity [25]. However, outside these periods of sexual receptivity, females are subject to extremely high levels of sexual harassment attributable to forced copulations by males, which in some natural populations have been shown to exceed one unsolicited mating attempt per minute [26]. Females incur significant costs through these forced matings, including reductions in foraging efficiency [27] and cross-generational reductions in offspring fitness [28]. Because natural rates of female multiple mating are typically greater in populations where males employ forced matings more frequently (due to concomitant changes in adult sex ratios, predation and other ecological factors) [29], levels of sexual harassment *and* multiple mating are likely to be inextricably linked in guppies. Consequently, any benefits enjoyed by multiply-mated females through consensual matings may be offset by the direct and indirect costs attributable to their higher overall mating frequencies.

**Figure 1:**
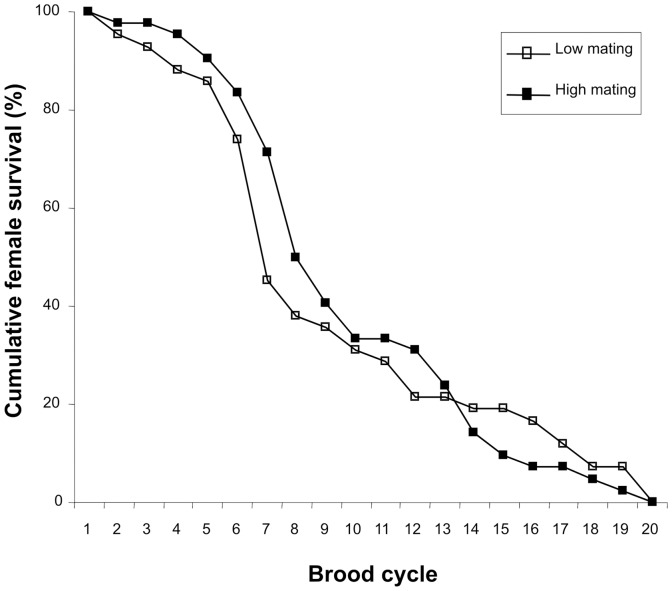
Cumulative survival curves for females assigned to high (filled boxes) and low (unfilled boxes) mating treatments.

In this study I examine how the overall level of multiple mating (lifetime number of mates) influences direct components of reproductive success in female guppies. Specifically, I examine patterns of female reproductive success over successive brood cycles that incorporate both the female's sexually receptive and non-receptive phases, from the onset of sexual maturity until death. This study therefore assesses the potential costs and benefits of multiple mating over a female's lifespan, thus testing whether the benefits of multiple mating documented early in a female's reproductive life [23] are traded against costs incurred subsequently [28].

**Figure 2:**
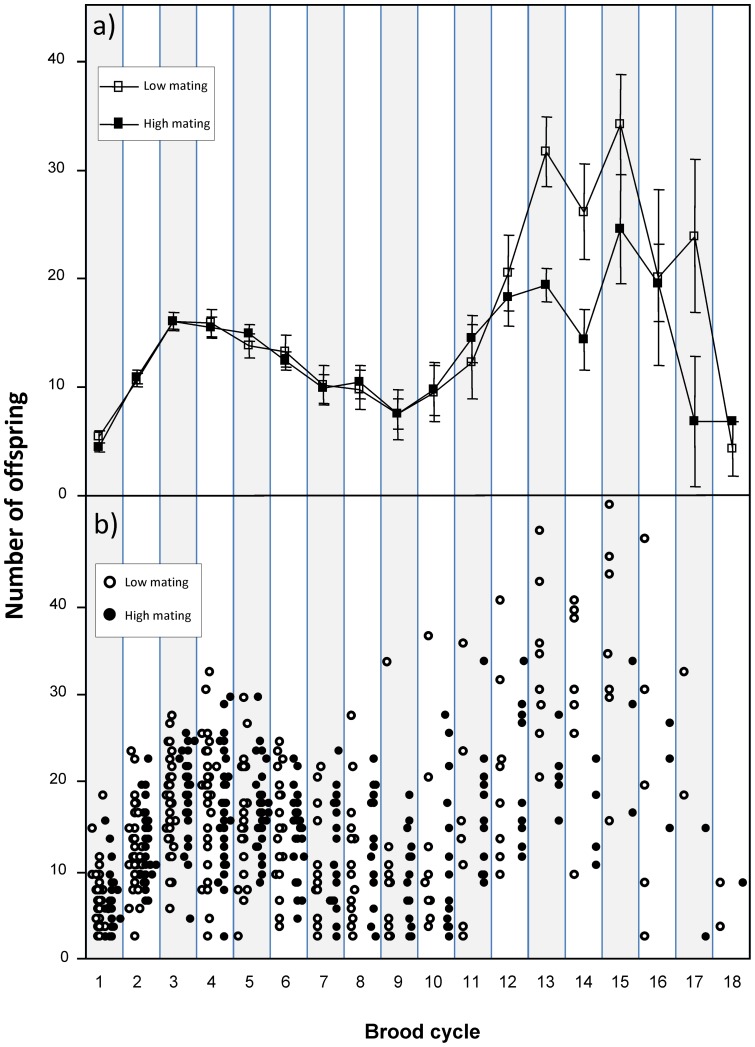
Number of offspring produced by females assigned to high and low mating treatments. (a) Mean ± SE number of offspring produced during successive brood cycles by females assigned to high (filled boxes) and low (unfilled boxes) mating treatments; (b) individual data points for brood sizes from females assigned to the high (filled circles) and low (unfilled circles) mating treatments. Note that where data points overlap in panel b, a ‘jitter’ function was used to displace the markers.

**Figure 3:**
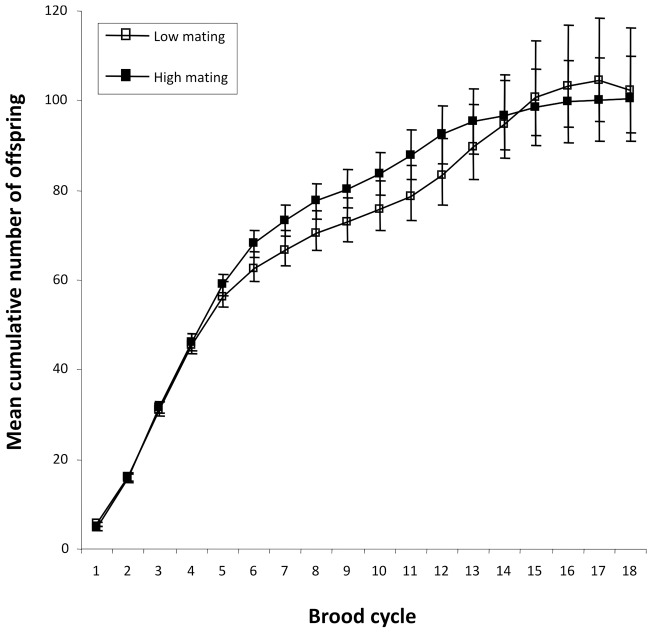
Mean ± SE cumulative number of offspring produced over the reproductive lifespan of females assigned to high (filled boxes) and low (unfilled boxes) mating treatments.

**Table 1:** Linear mixed-effects models testing the effect of mating treatment on (a) brood development time and (b) mean number of offspring produced by female guppies assigned to high- and low-mating treatments.

(**a**) **Brood development time**				
** Fixed effect**	***F*** **-value**	**df num**	**df den**	***P*** **-value**
Treatment	0.04	1	18.74	0.84
** Random effects**	**Var**	**SD**	***G***	***P*** **-value**
Female	4.75	2.18	13.5	<0.001
Brood cycle	5.39	2.32	5.4	0.01
Brood cycle*Treatment	1.86	1.36	2.3	0.06
Residual	46.45	6.81		
(**b**) **Mean number of offspring**				
** Fixed effect**	***F*** **-value**	**df num**	**df den**	***P*** **-value**
Treatment	1.72	1	11.69	0.21
** Random effects**	**Var**	**SD**	***G***	***P*** **-value**
Female	6.08	2.47	41.2	<0.0001
Brood cycle	35.76	5.98	11.5	<0.001
Brood cycle*Treatment	7.37	2.72	4.0	0.041
Residual	32.11	5.67		

## Materials and Methods

### Ethics statement

All animal work conducted as part of this experiment was done in accordance with the University of New South Wales's Animal Care and Ethics Committee (approval number ACEC 03/15).

### Origin and maintenance of fish and experimental overview

The fish used in this experiment were descendants of guppies caught from a feral population in the Alligator Creek River in Queensland, Australia. The experiment took place in a constant temperature room (set at 26°C) with lights set on a 12–12 h light-dark cycle. Virgin females (aged three months and matched for size by eye) were assigned at random to either a low (*n* = 42) or high (*n* = 42) mating treatment (LM and HM, respectively). Females assigned to the LM treatment interacted with a single (different) male for the first 14 d of each consecutive brood cycle until they died or ceased to produce any further offspring, while those assigned to the HM treatment interacted with two (different) males for the same duration and over the same period. Food levels (*Artemia* nauplii) were standardized so that LM treatments received two thirds of the food given to HM females while males were present in the tank.

### Mating treatments

At the start of the experiment, virgin females were placed individually into 2 L tanks (19×11×11 cm) with a gravel substrate, airstone and plastic pondweed for cover. On the following morning, either one or two males were added to the tanks according to the experimental treatment. In all cases, sexually mature males were chosen haphazardly (i.e. at random) to ensure that the ‘average phenotypes’ (size, colour, etc.) of males did not systematically differ between treatments. However, due to the size and duration of this experiment, it was not logistically feasible to photograph and analyse individual male phenotypes to explicitly test for such differences. In the LM treatment, a single non-virgin male was placed with the female and left for 14 days, while in the HM treatment two non-virgin males were added to each tank for the same duration. In this first reproductive cycle, the 14-day period encompasses both the female's sexually receptive phase (virgin females are sexually receptive for 4–5 days from first encountering male(s)) and non-receptive phase (i.e. days 5–14) [30]. At day 14, the males were removed from their respective treatments and returned to stock aquaria where they played no further part in the experiment. In both treatments, females were then left alone until they produced their first brood. In all cases, and without exception, the female tanks were monitored twice daily, seven days per week, to check for offspring. When offspring were produced, they were removed from the female's tank, counted and the date of parturition was recorded in order to measure brood development times (time in days from the addition of the male [s] until production of the brood). After broods were removed, half of the water from each tank was removed and replaced with clean, conditioned water. One day after parturition, new males were added to each tank (i.e. one in the LM treatment and two in the HM treatment) and left for a further 14 days. As with the initially virgin females, this 14 day period would have incorporated both the receptive (2–3 days post-partum) and non-receptive (days 3–14) phases of the female's reproductive cycle [30]. Females were again checked daily for offspring, which were removed and counted as described above, again noting the time in days taken to produce the brood. This process (males added 24 h after parturition; removed at day 14; offspring removed and counted; tank cleaned…) was repeated until the last of the females died (27 months after the commencement of the experiment). The experiment therefore generated data on the cumulative (lifetime) number of offspring, brood development times, the number of reproductive cycles and female longevity (survival) for 84 females assigned to the LM and HM treatments.

### Statistical analyses

Brood size (number of offspring produced in each consecutive brood) and brood development times (the mean time in days from introduction of the male [s] to the production of a brood in each consecutive brood cycle) were analysed with linear mixed-effects models using the lme4 package in R version 2.14.1 [31]. In each model, treatment (LM and HM) was fitted as a fixed effect, while female ID (1–84), brood cycle (1–18), and the interaction between brood cycle and treatment were modelled as random effects. Log-likelihood ratio tests (one-tailed) were used to assess the significance of random effects [32], which involved calculating the difference in log-likelihoods between a full model (including the term of interest) and a reduced model (where the term was excluded) and comparing the resultant *G* statistic (−2 times the difference in log-likelihoods between the two models) against a *χ* ^2^ distribution with 1 degree of freedom [33]. From these analyses, the significance of the brood cycle-by-treatment interaction was assessed to determine whether any observed effect of treatment depended on brood cycle number (i.e. female reproductive stage – a correlate of female age). For the analysis of brood development times, estimates exceeding 50 days were excluded from the analysis as these would most likely be due to females failing to produce offspring in the preceding brood cycle(s) [30]. However, the results were unchanged when these data were included in the analysis. Finally, independent samples *t*-tests were used to compare brood number (total clutches produced) and female lifespan (in days) between LM and HM treatments.

## Results

Four of the original 84 females (two from each treatment) never produced offspring and were therefore excluded from the analyses of mean brood size and brood development times (below). There was no significant difference between treatments in the overall number of broods that females produced (mean number of broods ± SE: LM group  = 7.33±0.77; HM group  = 7.90±0.64; range 1–18; *t*_82_ = 0.57, *P* = 0.57). On average, females lived for 388.1 days from the start of the experiment (range: 185–821), and survival times did not differ significantly between treatments (mean number of days that female lived from the start of the experiment ± SE: LM group  = 381.25±26.43; HM group  = 394.95±23.28; *t*_78_ = −0.39, *P* = 0.70; Fig. 1).

The linear mixed-effects models (Table 1a & b) revealed no overall effect of mating treatment on either brood development times (mean number of days ± SE: LM  = 31.32±0.40; HM  = 31.51±0.46) or the mean number of offspring (total number of offspring produced in LM  = 4192 offspring; HM  = 4117; mean brood size ± SE: LM  = 13.61±0.53; HM  = 12.40±0.36 Fig. 2a). Thus, summed over a female's lifespan, there was no significant difference in the total number of offspring produced by females assigned to the LM and HM treatments (mean ± SE total number of offspring produced: LM  = 99.81±13.70; HM  = 98.02±9.32; *t*_82_ = 0.11, *P* = 0.91; see Fig. 3 for mean cumulative number of offspring produced by females assigned to the LM and HM groups). Nevertheless, there was a significant interaction between brood cycle and treatment (see Table 1b) because LM females produced significantly higher numbers of offspring later in life (brood cycle 13–18; see Fig. 2a). This interaction was largely attributable to an increase in offspring production in the LM group during brood cycle 13–15 which was not evident in the HM group, although it is important to note that this interaction arose during the latter phases of the experiment when sample sizes were reduced and variances were correspondingly high (see Fig. 2b for individual data points).

## Discussion

This study suggests that female guppies descended from the Alligator Creek population enjoy no net direct benefits of mating with multiple males over their lifetime, thus contrasting with results presented previously for fish descended from a Trinidadian population, where females enjoyed significant direct and indirect benefits of multiple mating, including an increase in mean brood size [23], [25]. In previous work, the benefits of multiple mating were recorded early in life (equivalent to brood cycle 1 in the present study), while in the present study mean brood sizes were almost identical at brood cycle 1 (Fig. 2). While this difference between studies may reflect the different population origins of the guppies used, it may also reflect important differences in experimental design. In particular, the present study systematically varied female mating frequency (i.e. male number) over the course of the females' sexually receptive and unreceptive phases in each brood cycle. Thus, benefits enjoyed by multiply-mated females during their receptive phase may have been offset by costs incurred as a consequence of elevated sexual harassment during their unreceptive phase. Although the present design makes it impossible to tease apart these costs and benefits, previous work has shown that females incur direct foraging costs as a consequence of sexual harassment, which in turn is thought to impact on their fecundity [27]. The attempts to standardize food intake between LM and HM treatments may not have been sufficient to counteract such effects.

This study focused on direct components of female fitness and related these to differences in lifetime levels of multiple mating. However, lifetime reproductive success (LRS) captures only *direct* components of a female's fitness, and ignores the known cross-generational (*indirect*) fitness effects attributable to mating with multiple males/mating frequency [23]–[25]. When exploring how mating frequency influenced female fitness in *Drosophila melanogaster*, Priest et al. [13] found that reductions in female lifetime reproductive success were offset by benefits enjoyed by their female offspring. Thus, frequent mating can generate cross-generational fitness trade-offs in which maternal fitness is traded off against offspring fitness [13]. Recent work has also explored cross-generational effects of mating in guppies, where the level of sexual harassment was experimentally manipulated whilst holding the level of multiple mating constant across a female's lifespan [28]. In that study [28], there were no overall direct costs of male sexual harassment, but females subjected to high levels of sexual harassment produced offspring with impaired fitness. Interestingly, Gasparini et al. [28] also reported a significant interaction between brood cycle and mating treatment (in their case the level of sexual harassment independent of multiple mating), supporting the conclusion that any costs of sexual harassment are confined to the latter phases of a female's life. However, in the present study, the interaction between treatment and brood cycle was largely driven by an *increase* in brood size later in life in the LM treatment rather than a cost expressed by HM females (see Fig. 2a). The biological relevance for this increase in brood size later in life in LM females is unclear as we presently lack information about natural lifespan in the focal population used for this study. Notwithstanding this, an interesting direction for future work will be to combine the methods employed in the present study (which systematically varies both mating frequency and male number) with those employed by Gasparini et al. [28] to determine whether the indirect benefits of multiple mating [23], [24] balance the cross-generation fitness costs of male sexual harassment [28].
